# Editorial: Adopting New Technologies in Sports Marketing

**DOI:** 10.3389/fspor.2021.777841

**Published:** 2021-10-27

**Authors:** Hyun-Woo Lee, Natasha T. Brison, Heetae Cho, Do Young Pyun, Vanessa Ratten

**Affiliations:** ^1^Department of Health and Kinesiology, Texas A&M University, College Station, TX, United States; ^2^Department of Physical Education and Sports Science, Nanyang Technological University, Singapore, Singapore; ^3^School of Sport, Exercise and Health Science, Loughborough University, Loughborough, United Kingdom; ^4^Department of Management, Sport and Tourism, La Trobe University, Melbourne, VIC, Australia

**Keywords:** sport, marketing, technology, media, communication

New technologies have revolutionized nearly every aspect of human existence, impacting how companies commercialize goods and services (Grewal et al., 2020). More radical innovations are quickly emerging on the heels of the present generation of innovations, such as greater computing capacity, the Internet, social media, and mobile devices and applications. These technological advances are exerting profound effects on the practice of marketing (Gillpatrick, 2019). Specifically, applications and solutions of technological innovations compel marketers to pre-emptively test their unpredictable outcomes and effects (Kumar and Ramachandran, 2018).

New technologies are also rapidly revolutionizing the sport industry landscape, providing sport marketers—from small sport retailers to top sport brands—with novel methods to reach customers. For example, technological innovations, such as augmented reality (AR; Goebert and Greenhalgh, 2020), sport wearable technology (Kim and Chiu, 2019), the meteoric rise of mobile commerce and smartphones (Kim et al., 2017), and virtual reality (VR; Uhm et al., 2020), have driven major changes in the sport industry and influenced how sport markets are managed. These emerging technologies are fundamentally transforming sport consumer experiences and expanding the boundaries of sport marketing research.

Studies of new technology in sport, however, have been largely neglected in the greater body of sport marketing literature with minimal attention to their merits or demerits (Ratten, 2020a). In fact, studies on new technologies in sport marketing have been mainly published in journals of other disciplines rather than those dedicated to sport marketing (e.g., Kim and Ko, 2019; Goebert and Greenhalgh, 2020). Greater scholarly attention to adopting new technologies in sport marketing is needed to better understand and embrace these innovations in the sports industry. The COVID-19 pandemic has shown us that there is a need for sport technology, particularly when cities and countries have lockdowns that restrict the ability of individuals to play and watch sport (Ratten, 2020b).

This Editorial and four articles introduce and add to the growing body of knowledge regarding the development and use of technology in sport marketing.

Two of the articles contribute new knowledge regarding the theme of new media and communication tools. Particularly in the era of COVID-19, these two articles underscore how these media technologies offer sport consumers a sense of safety while simultaneously creating the impression of exercising and spectating a match in a real stadium despite being at home. Explaining how the COVID-19 pandemic has affected athletic events and communications in sports, Zaborova et al. present telecommunication technologies commonly utilized to enable children to perform basic gymnastic exercises at home. Specifically, Zaborova et al. explore how virtual technology can be employed in the field of education. Similarly, Lupinek et al. provide a virtual reality (VR) In-Game Advertising (IGA) congruity framework that seeks to help marketers make well-reasoned IGA development decisions through strategic choices. According to Lupinek et al., the centralized VR IGA congruity framework not only enhances brand awareness but also imbues participants with a favorable mindset toward the IGA, ultimately increasing sales activation. In this respect, we can assume that VR is emerging as a powerful marketing tool by eliciting novel and enjoyable consumer experiences. The studies of Zaborova et al. and Lupinek et al. showed that new media technologies, such as virtual and augmented reality, are expected to continue advancing and see active application within the sports industry.

The other two articles include an investigation of social media engagement for women's sport and a valuation of sport media service. Pegoraro et al. examine how broadcasting impacts social media engagement with respect to game day WNBA team account posts, discussing how digitally streaming live sport events offers opportunities for women's sport and niche sport that lack television contracts and/or have limited resources to support their own digital streaming services. Furthermore, Pegoraro et al. consider advances in technology and changes in consumer preferences and explore how behaviors have led to numerous new broadcast options for sport leagues to distribute their contents directly to the sport market and consumers by expanding a range of choices. In addition, based on the unified theory of acceptance and the use of the technology model, Huettermann et al. build and empirically test a conceptual model of factors leading people to accept a novel sport club video service. In particular, Huettermann et al. assessed potential users' willingness to pay for a total sport club video service solution from production to distribution. Huettermann et al. provide practical implications for forward-looking developers by pinpointing relevant factors that are persuasive to sport managers willing to adopt new technologies.

The four studies that contribute to this Research Topic vary in their inquiries into existing knowledge, such as what kinds of new technologies are presently being adopted in sport marketing, how marketing strategies are implemented through new technologies, and what factors have caused these new technologies to be applied in the sport market. These studies do not inclusively mention every new technology in the sport industry as, for example, artificial intelligence (Davenport, 2018) and robotics (Mende et al., 2019) are currently being introduced into the sport marketing field. However, it is expected that this Research Topic serves as a cornerstone to attract readers' attention in that it provides a variety of knowledge about technologies that have thus far received little attention among sport marketing scholars.

In conclusion, our examination of this Research Topic has resulted in a novel collection of articles that further our knowledge of new technologies in sport marketing. The wealth of contents presented within this Research Topic is illustrative of the growing interest in the greater area of new technologies in sport marketing and thus provides a stepping stone to further innovative studies. We hope that the articles selected here will act as a potent stimulus for research in this exciting area and encourage the exploration of future topics that may build upon the findings presented in these papers.

## Author Contributions

All authors listed have made a substantial, direct and intellectual contribution to the work, and approved it for publication.

## Conflict of Interest

The authors declare that the research was conducted in the absence of any commercial or financial relationships that could be construed as a potential conflict of interest.

## Publisher's Note

All claims expressed in this article are solely those of the authors and do not necessarily represent those of their affiliated organizations, or those of the publisher, the editors and the reviewers. Any product that may be evaluated in this article, or claim that may be made by its manufacturer, is not guaranteed or endorsed by the publisher.
